# Renal Manifestations of Tuberous Sclerosis Complex

**DOI:** 10.15586/jkcvhl.2020.131

**Published:** 2020-08-27

**Authors:** Nikhil Nair, Ronith Chakraborty, Zubin Mahajan, Aditya Sharma, Sidharth K. Sethi, Rupesh Raina

**Affiliations:** 1 Department of Chemistry, Case Western Reserve University, Cleveland, OH, USA;; 2 Akron Nephrology Associates, Cleveland Clinic Akron General, Akron, OH, USA;; 3 Northeast Ohio medical University, Rootstown, OH, USA;; 4 Pediatric Nephrology, Medanta, The Medicity, Gurgaon, India;; 5 Department of Nephrology, Akron Children’s Hospital, Akron, OH, USA

**Keywords:** angiomyolipoma, autosomal polycystic kidney disease, renal cystic disease, tuberous sclerosis, Von Hippel–Lindau disease

## Abstract

Tuberous sclerosis complex (TSC) is a genetic condition caused by a mutation in either the *TSC1* or *TSC2* gene. Disruption of either of these genes leads to impaired production of hamartin or tuberin proteins, leading to the manifestation of skin lesions, tumors, and seizures. TSC can manifest in multiple organ systems with the cutaneous and renal systems being the most commonly affected. These manifestations can secondarily lead to the development of hypertension, chronic kidney disease, and neurocognitive declines. The renal pathologies most commonly seen in TSC are angiomyolipoma, renal cysts, and less commonly, oncocytomas. In this review, we highlight the current understanding on the renal manifestations of TSC along with current diagnosis and treatment guidelines.

## Introduction

Tuberous sclerosis complex (TSC) is an autosomal dominant inherited disease, characterized by lesions that involve multiple organs of the body and variable clinical manifestations. Although TSC shows dominant inheritance, 60–70% of patients are sporadic cases due to *de novo* mutation. TSC has an estimated incidence of ~1 in 5800 births, with prevalence rates of 1 in 7000–20,000. Currently, there are approximately 2 million people who are believed to be affected by TSC worldwide, with approximately 40,000–80,000 patients in the United States (1). In spite of its relative rarity, the identification of TSC and its comorbid conditions are crucial for mitigating long-term negative outcomes in these patients. While TSC is more familiar to pediatricians, adult providers are much less trained to identify and treat this condition. Effective understanding is crucial for ensuring a stable condition throughout the patient’s lifetime.

TSC is characterized by benign tumors that can develop on the brain, skin, heart, lungs, kidneys, and liver (1, 2). Symptoms and manifestations of this condition include facial/topical angiomyolipomas (AMLs), cardiac rhabdomyomas, brain subependymal giant cell astrocytoma (SEGA), lymphangiomyomatosis, retinal astrocytic renal AML, and renal cell carcinoma (RCC). In addition to these conditions, neurologic conditions are especially prevalent in the form of mental stunting, autism, and epilepsy. Renal manifestations, in particular, are important to monitor, as they are found to be the number one cause for mortality in TSC patients, particularly due to malignant renal AMLs and RCC in younger patients (3). While neurological, renal, and cutaneous manifestations are the most common, the significance and severity can vary across individuals. Furthermore, the onset of TSC can take several years to manifest. As a genetic condition, TSC is a lifelong condition and requires regular monitoring of patients to ensure that tumor growth or new symptoms are identified as early as possible (3, 4). While this manuscript focuses on renal manifestations, Table 1 outlines the frequency and symptoms of TSC manifestations in other systems of the body. The multiorgan onset of conditions necessitates the cooperation of multiple specialty providers to get the best possible outcomes for the patient.

**Table 1:** The manifestations of tuberous sclerosis complex and the percentages of distribution

Type	Percentage	Description
Cutaneous	90	Skin lesions in all ages
**Renal**	**80**	**#1 Morbidity/Mortality: kidney function, hemorrhage, cysts, carcinoma**
Neurological	70–90	**#2 Morbidity & Mortality: epilepsy, seizures, autism, mental retardation**
Ophthalmologic	30–50	Retinal hamartoma, bilateral and multiple, no visual impairment
Pulmonary	40	Cysts, smooth muscle, mainly adult females, reduced pulmonary function
Cardiac	80	Rhabdomyoma, mainly fetus and newborn, cardiomegaly, murmurs

Adapted from Schepis C. The tuberous sclerosis complex. Dermatol Cryosurg Cryother. 2016;615–17. (1).

**Figure 1:**
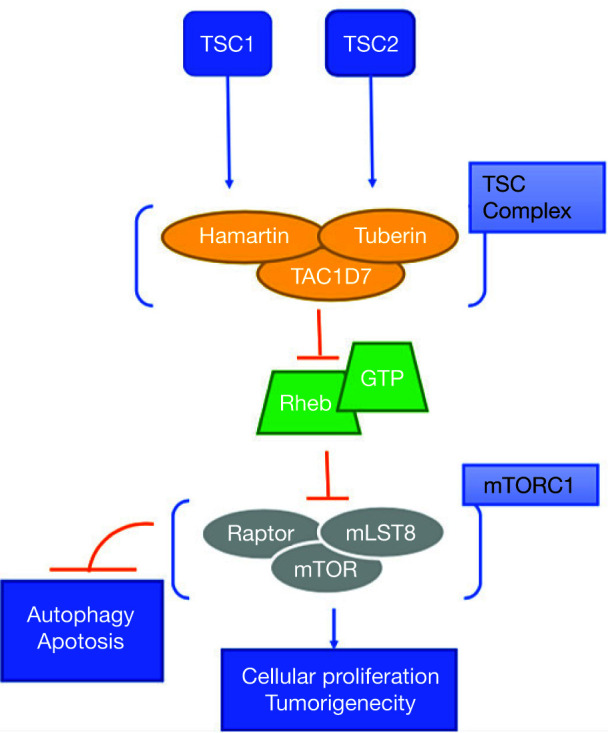
Normal downstream pathway of TSC1 and TSC2, and its gene products. These proteins are involved in the regulation of cell growth through the phosphatidylinositol 3-kinase signaling pathway (PI3K), an inhibitor of the mammalian target of rapamycin (mTOR). TSC1: tuberous sclerosis complex 1; TSC2: tuberous sclerosis complex 2; Rheb: Ras homolog enriched in brain; GTP: Guanosine triphosphate; Raptor: regulatory-associated protein of mTOR; mLST8: target of rapamycin complex subunit LST8; mTOR: mammalian target of rapamycin.

The genetic mechanism behind this condition results from a mutation to either the *TSC1* gene on chromosome 9 or the *TSC2* gene on chromosome 16 (5, 6). Two-thirds of the cases occur sporadically and are caused by new mutations. Within families with multiple afflicted members, about half of the patients have shown linkage to locus 9q34 with the other half to 16p13 (7). Physiologically, these proteins are involved in the regulation of cell growth through the phosphatidylinositol 3-kinase signaling pathway (PI3K), an inhibitor of the mammalian target of rapamycin (mTOR). Mutations in the genes encoding these proteins cause the permanent activation of the mTOR pathway, leading to uncontrolled cellular proliferation and formation of hamartomas in various organs of the body. Genetic mutation in TSC can lead to the deletion, rearrangement, or inactivation of the *TSC1* or *TSC2* genes. While hereditary germline mutations are observed, majority of TSC patients (70%) express the condition through somatic mutations (8). Familial transmission, more commonly, has less severe manifestations of the disease, at times not even fulfilling all the diagnostic criteria of TSC. Therefore, these hereditary manifestations mostly show mutations to *TSC1* (7). Figure 1 displays the downstream pathway of *TSC1* and *TSC2* dysregulation.

The *TSC1* gene is composed of 23 exons, with 21 encoding hamartin (130 kDa). The *TSC2* gene consists of 42 exons, with 41 encoding tuberin (180 kDa). Mutation detection efforts in TSC patients have found mutations in approximately 85% of the patients studied (9, 10). Table 2 identifies recommendations from the 2012 consensus conference on TSC that a physician can follow if TSC is suspected in a patient. In order to clinically confirm the presence of TSC, major and minor features need to be present, as defined by the 2012 International TSC consensus conference, as shown in Table 3.

**Table 2:** Recommendations for physicians for potential TSC diagnosis.

**Genetic**
Offer genetic testing for the families in situations where TSC diagnosis cannot be confirmed clinically.
Obtain genetic family history in order to identify other members who may be at risk.
**Kidney**
Order MRI to screen for the presence of angiomyolipoma and renal cysts in the abdominal region.
Screen for hypertension from clinical blood pressure readings.
Monitor proper renal function by determination of glomerular filtration rate (GFR).

Adapted from Krueger DA, Northrup H, International Tuberous Sclerosis Complex Consensus Group. Tuberous sclerosis complex surveillance and management: Recommendations of the 2012 International Tuberous Sclerosis Complex Consensus Conference. Pediatr Neurol. 2013;49(4):255–65. 10.1016/j.pediatrneurol.2013.08.002. (11).

TSC, Tuberous sclerosis complex; MRI, magnetic resonance imaging.

**Table 3:** Necessary features for diagnosing TSC.

**Major features**	
Dermatological	hypomelanotic macules (≥3, *at least 5 mm diameter*), angiofibroma (≥*3*) ungual fibromas (≥*2*) shagreen patch
Ophthalmological	Multiple retinal hamartomas Cortical dysplasias^*^
Neurological	Subependymal nodules Subependymal giant cell astrocytoma
Cardiothoracic	Cardiac rhabdomyoma
Pulmonary	Lymphangioleiomyomatosis Angiomyolipomas (≥2)
Renal	Angiomyolipomas (≥2)
**Minor features**	
Dermatological	“Confetti” skin lesions Dental enamel pits (>3) Intraoral fibromas (≥2)
Renal	Multiple renal cysts
Endocrine	Nonrenal hamartomas

Adapted from Krueger DA, Northrup H, International Tuberous Sclerosis Complex Consensus Group. Tuberous sclerosis complex surveillance and management: Recommendations of the 2012 International Tuberous Sclerosis Complex Consensus Conference. Pediatr Neurol. 2013;49(4):255–65. 10.1016/j.pediatrneurol.2013.08.002. (11). TSC, Tuberous sclerosis complex.

The TSC genotype is one the strongest predictors of renal manifestation (12). *TSC2* mutant patients typically display increased incidence and severity of angiomyolipomata and cysts compared to *TSC1* mutant patients (11). While severe renal disease outcomes can occur in *TSC1* mutants, these patients are significantly less likely to exhibit severe renal manifestations than those with *TSC2* mutation. Generally, patients who have no identified mutations (NIM) show syndromic manifestations that are less severe than patients with known mutations, especially in terms of cognitive and seizure symptoms. Furthermore, it has been observed in a systemic analysis by Seyam et al. that there is a higher frequency of AML manifestations in patients with NIM than those with *TSC1* mutations. These differences have not been observed between NIM and *TSC2* (13). These findings suggest that the renal manifestation of patients with NIM most closely resemble that of *TSC2* instead of *TSC1*. This could be due to a defect in *TSC2* that’s transcriptional, translational, or post-translational in nature. In addition, these patients warrant a closer examination of the *TSC2* sequence and the effect that protein activity may have in NIM patients. The correlation between gender and renal manifestation is seemingly only significant for AML appearance (14). Female patients display a higher frequency of AMLs than males potentially due to the presence of increased estrogen and progesterone receptors on tumors. In addition, a large, double-blind study showed that females have an increased incidence of adverse outcomes from angiomyolipomata (15, 16). Furthermore, renal AMLs are found in larger quantities and have larger volumes in women, compared to men. This suggests a hormonal effect on AML development and growth; this can also occur in prepubertal girls, who have higher estradiol levels than boys (17).

## Pathophysiology

### Angiomyolipoma

Renal AMLs are the most common benign tumors observed in TSC patients. The presentation of AMLs has been reported in 75–85% of TSC patients with renal lesions and in 49–60% of TSC patients, overall (3, 18). AMLs derive from the PEComa family of tumors, exhibiting immunoreactivity for both melanocytic markers (HMB-45 and melanin-A antibodies) and smooth-muscle markers (actin and desmin). AMLs are composed of abnormal blood vessels, immature smooth-muscle cells, and fat cells, although the proportions can vary between lesions within the same kidney (15). Presumably, this deficiency disrupts the integrated control of cell growth, leading to the AML (6). These tumors then arise by clonal proliferation of epithelioid cells distributed around blood vessels and, in patients with TSC, include renal AMLs and pulmonary lymphangioleiomyomatosis (LAM) (19). A neural crest cell origin has been proposed based on the co-expression of melanocytic and smooth cell markers, with two cell lineages known to arise, at least in part, from neural crest cells (20, 21). Re-expression of *TSC2* in an AML has shown to result in the maturation of AMLs into lymphatic endothelial cells (21). This has led many investigators to believe that AMLs do not actually arise from neural crest cells; instead, they may arise from the lymphatic endothelium (22). Knocking-out *TSC2* and *Cdkn2a* in the murine epithelial cell line gave rise to lesions that had some AML findings; however, an important caveat for these findings was that *Cdkn2a* encodes two proteins, p16 and p14ARK, and is associated with melanoma (23). In some studies, mesenchymal stem-cell-like cells have been identified in AMLs, and pericytes have been strongly implicated as the cell of origin for angiomyolipomata (4). These recent discoveries are interesting given the prevalence of vascular aneurysms associated with TSC that arise in angiomyolipomata in the aorta and the brain (24–26). The lesions in AML express smooth-muscle actin and melanocyte markers, such as gp100, a splice variant of Pmel17, and even melanin A. Expression of these melanocyte-associated genes likely results from the mTOR-mediated increase in the microphthalmia (MITF/TFE) family transcription factor activity (27). This increased MITF/ TFE transcription factor activity has caused confusion between TSC-associated PEComas, the much more aggressive RCCs, and the PEComas associated with translocations involving the TFE3 or TFEB transcription factors of the MITF/TFE family (28).

### Cystic disease

Renal cysts have been observed in 14–32% of the TSC population and have predominately two types of manifestations. The most common presentation is either single or multiple small lesions, rarely symptomatic but in unison in their histology. A less common cystic manifestation of TSC coexists with polycystic kidney disease, which indicates a poor prognosis for survival. The mTOR pathway is strongly associated with primary cilia-associated cystogenesis. Investigations of *TSC1-* or *TSC2*-associated renal cystic disease in mice have shown that cystogenesis can be attributed to specific nephron segments. Furthermore, all tubular segments have been found to be involved in murine TSC cyst formation (29). Incongruous findings have been attributed to tubular epithelial dedifferentiation, a repair/regeneration process that recapitulates renal development (30). In mouse models studied, there seems to be significant elevation of cyst mTORC1 activity (phospho-S6 expression) but a very low percentage of cells exhibiting loss of TSC expression (27). The somatic mutation (second hit) dogma of TSC renal disease is difficult to reconcile with the early investigations that failed to find loss of heterozygosity in a majority of cysts, indicating that TSC locus integrity is preserved in most renal cysts (31). The low percentage of cysts with loss of heterozygosity is similar to human TSC renal cystic disease, because human cysts also continue to express tuberin and hamartin, in contrast to the mechanism for angiomyolipomata, which show an inactivating mutation and loss of heterozygosity, resulting in the loss of tuberin staining (32). The low percentage heterozygosity loss has also been seen in *PKD1*-associated autosomal dominant polycystic kidney disease (ADPKD), suggesting that cystic disease may possibly represent a novel disease mechanism (29).

### Autosomal dominant polycystic kidney disease

ADPKD accounts for fewer than 2% of TSC cases (33, 34). The phenotypic intersection between TSC and ADPKD cases indicate the existence of a functional relationship between the respective genes that have been recently identified as the mTOR signal pathway (35). Under normal conditions, polycystin-1 and the *TSC1/TSC2* tumor suppressor genes inhibit the function of mTOR, contributing to the suppression of cell replication at the G1-phase and subsequently resulting in apoptosis. The tuberin protein traffics polycystin-1 to the plasma membrane, which may be the fundamental basis for their genetic collaboration (36). TSC may therefore coexist with ADPKD in the same patient as a by-product of concurrent germline deletion of both *PKD1* and *TSC2* genes (37). Approximately, 75% of TSC patients are from the pediatric age range (38).

Patients with an omission of both the *PKD1* and *TSC2* gene advance to a premature and radical onset of PKD. The disease aggression might be a consequence of transformed gene expression by microRNAs as a product of convergent transcription of genes on the unaffected allele (39). Cyst formation in TSC patients with ADPKD ensues through a two-hit tumor suppressor mechanism, with loss of heterozygosity, particularly in the epithelial cells lining the cyst wall (30, 40). ADPKD patients with TSC usually present with abdominal distension or secondary hypertension and in some cases with hematuria secondary to the rupture of the cysts (40). These patients have an advanced risk of progression to end-stage renal disease ESRD beginning around 30 years of age (41). In a study by Sampson et al., the authors demonstrated that cases with deletions in *TSC1/PKD2* have enlarged cystic kidneys during infancy or childhood with features of advanced ADPKD at the time of diagnosis (42).

### Phenotypical overlap of VHL to TSC

While TSC and *Von Hippel-Lindau disease* (VHL) are caused by mutations in different genes, they show phenotypical and possible pathophysiological similarities. Both conditions are autosomal dominant disorders, characterized by solid and cystic renal pathologies due to mutations in the tumor suppressor genes. Both syndromes are also characterized by highly vascularized tumors, although RCCs are rarer in TSC than in VHL. The phenotypic expression of these condition ensues when there is an inherited deactivating mutation in the *TSC1*/*TSC2* gene for TSC or the *VHL* gene for VHL syndrome, combined with an additional somatic mutation in the affected locus. Even though TSC and VHL are the end result of two distinct mutations, they have certain resemblances with regard to their molecular pathogenesis (43). TSC and VHL disease are both diverse tumor suppressor syndromes that have some common renal phenotypic features, including cysts and vascular tumors such as RCC. These resemblances may be the terminal consequences of intersection in signaling and gene expression effectors, such as primary cilia, vascular endothelial growth factor (VEGF), and β-catenin. The neoplasms in VHL display excessive production of messenger ribonucleic acid specific to hypoxia-inducible factor (HIF)-dependent growth factors, such as VEGF, and this additional activity of VEGF and other hypoxia-inducible genes contribute to the development of neoplasms in TSC (43). TSC is characterized by unwarranted production of HIF factors while VHL includes suppression of proteasome-facilitated elimination of HIF (44). Tuberin independently regulates VEGF activity via mTOR-dependent and independent mechanisms. Loss of tuberin accelerates upregulation of VEGF, which elucidates the exceedingly vascularized nature of these neoplasms seen in TSC (44). Both diseases have a concrete association with the development of renal cystic disease, and it has been observed that renal cysts are attributed to mutations in the cilia and the associated proteins (25). There is additional evidence to show that the coordinated activity of tuberin, polycystin, and mTOR, and its dysregulation has contributed to the expansion of renal cysts (45). Loss of regulatory properties of β-catenin is additionally linked to the renal cystic changes in VHL and TSC patients (46, 47). Renal complications in these patients mandate follow-up with regular physical exam, radiological imaging, and laboratory tests. Identification and appropriately diagnosing each condition are paramount to ensuring the best possible outcomes for patients. Furthermore, increased information regarding the signaling pathways has commenced clinical trials for various pharmacological therapies (48–51).

### Malignant tumors

#### Malignant AMLs and RCCs

In spite of the characteristic presence of benign tumors, the risk of malignant tumors is relatively low in TSC patients. Malignant tumors are seen in approximately 3% of patients and can be diagnosed as RCC or malignant epithelioid AML. These tumors develop at an average age of 36 years, 20 years earlier than the average age of onset (52). RCC accounts for 2–3% of all adult malignancies and is the most common type of kidney cancer (52). There are a significant variety of histologies with regard to RCC in TSC. The two main categories include clear cell RCC (ccRCC, ~85%) and nonclear cell RCC (nccRCC, ~15%). Both limited pathological data and overlapping physiology with fat-poor AMLs have inhibited the ability to elucidate the entire picture. Few RCCs in TSC patients show simultaneous expression of melanocytic and renal tubular markers and as such contribute to the heterogeneity of the tumors seen (53). Given the absence of the VHL gene in the majority of TSC patients, these types of RCCs develop independently of the canonical pathways (53). In TSC, drivers of RCC include loss-of-function mutations of *TSC1*/*TSC2* genes that lead to the inactivation of the TSC signaling pathway (54). Malignant AMLs also have varied presentations in TSC, leading to confusion during diagnosis between epithelioid AMLs and RCC. The use of immunocytochemical staining specific for cytokeratin and HMB-45 can help distinguish between the two (54).

#### Clinical manifestation

AMLs are detectable at a younger age in patients with *TSC2* mutations compared to patients with *TSC1* mutations (13 years vs. 24 years), and intervention is more often needed in patients with *TSC2* mutations compared to those with *TSC1* mutations (27% vs. 13%) (17). Throughout the first few years of life, there is a progressive enlargement of the tumors with expedited growth during adolescence and/or early adulthood, followed by a decreased growth in 30% of elderly patients. AMLs are the primary reason for the morbidity and mortality seen in TSC patients, in spite of many being asymptomatic (17). When present, clinical manifestations include hemorrhage, leading to hematuria and loss of kidney function, with a greater risk of hemorrhage in tumors larger than 30 mm in size.

Symptomatic kidney cysts are seen in 30–50 % of TSC patients with renal manifestations. These are more frequently seen in patients with *TSC2* mutations, compared to those with *TSC1* mutations (55). AMLs that are sporadic and less than 2 cm are of little clinical relevance and are not associated with decline in renal function (56). When AMLs are numerous and actively growing, the risk of renal dysfunction is increased, especially in approximately 3% of TSC patients with a contiguous gene syndrome affecting the *TSC2* and *PKD1* genes (57). Recommendation on treatment and monitoring of different sized AML by Dickinson et al. is illustrated in Table 4 (19). In these cases, renal cysts can develop early with the clinical course of renal function resembling that of ADPKD patients with ESRD in the second or third decade of life (58).

**Table 4:** Diagnosis and treatment of angiomyolipoma.

Size	Characteristic	Treatment/monitoring
Small (<4 cm)	Generally asymptomatic,	Should be monitored on a yearly basis but elective treatment not necessary.
Medium (4–8 cm)	Highly variable in behavior with the propensity to either grow quickly or remain benign.	Due to their variable nature, more frequent images should be taken. To preserve renal function, preventative treatment by mTOR inhibitors should be taken.
Large (>8 cm)	Most likely to be symptomatic; largest risk for malignancy and vascularization, leading to increased risk of hemorrhage.	Once observed, elective treatment should be taken immediately to reduce the chance of symptoms and complications developing. If no immediate risk of hemorrhaging presents itself, mTOR inhibitors should be taken to reduce the mass and the volume of the lesion.

Adapted from Dickinson M, Ruckle H, Beaghler M, Hadley HR. Renal angiomyolipoma: Optimal treatment based on size and symptoms. Clin Nephrol. 1998 May;49(5):281–6. (21).

mTOR, mammalian target of rapamycin.

The most common clinical manifestations of symptomatic renal AMLs are the various forms of hemorrhage (intratumoral, hematuria, or retroperitoneal hemorrhage) (59). The potential of developing these symptoms increases with increase in the size of the AML. Renal AMLs > 4 cm are more likely to grow and develop microaneurysms (43, 60). Likewise, the risk of significant hemorrhage is related to the degree of vascularity, the size of the AML, and the size of aneurysms within the AML. Increased vascularity and/or aneurysm size (≥5 mm) are associated with an increased risk of rupture (24). Furthermore, the rate of growth should be monitored, with the most risk of hemorrhage in those enlarging at a rate of >30 mm (61).

The risk of bleeding can also increase during pregnancy due to hormonal changes and/or increased blood volume (62). Hormonal imbalances are potential causes for the increased rate of AML growth from infancy to adulthood. In contrast to the uniformly benign prognosis of classic renal AMLs, epithelioid variants may undergo malignant transformation, although this is quite infrequent. Malignant transformation can be manifested by both local recurrence and/or distal metastases. The relative risk is greater in patients with TSC, but most patients with malignant transformation have sporadic renal AMLs (63, 64).

In a large-scale study of 164 pediatric patients with TSC, who underwent serial renal ultrasonography or computed tomography (CT), the authors found that the rate of renal AML increased up to an excess of 50% after the age of five with no renal AMLs in the first year of life (65). In another longitudinal study involving 60 children, rates of renal AMLs increased from 48% in boys and 60% in girls at a mean age of 6.9 years to 76% in boys and 83% in girls at follow-up at a mean age of 10.5 years (33). By the time these patients reached adulthood, 33% of the AMLs stopped growing (61).

**Figure 2:**
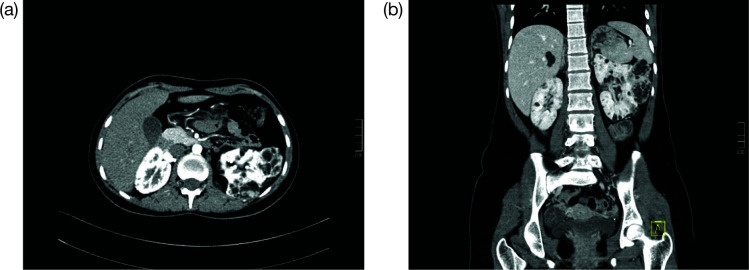
Contrast enhanced CT images of abdomen in axial (a) and coronal (b) planes. This is the case of a 16-year-old female with tuberous sclerosis undergoing treatment with everolimus. The CT scan shows right heminephrectomy and an enlarged left kidney with multiple cysts and angiomyolipomas.

The primary methods to diagnose the presence of AMLs are imaging technology, ultrasound (US), and magnetic resonance imaging (MRI) in the abdominal region (65). An example of a positive image is shown in Figure 2. However, cautious interpretation is warranted when considering these US data, as US often cannot detect fat-poor angiomyolipomata, with approximately a third of patients demonstrating fat-poor lesions. Continued monitoring of renal AMLs is crucial, as they exhibit continued growth and patients may develop aneurysms as the AMLs enlarge. Increased size also leads to an increased risk of bleeding; AMLs > 3 cm and aneurysms ≥ 5 mm are associated with an increased risk of severe bleeding (61).

### Fat poor AML

Nearly 5% of TSC-associated AMLs observed are fat-poor in nature. Containing an average of 4% fat by mass, these lesions often have similar tissue density as renal parenchyma, have the same echotexture as the kidneys, and are often not identified on US. The International Consensus Guidelines by the 2012 TSC consensus conference recommends the use of MRI for screening, as they are often missed in US (66). In addition, at least half of the patients affected by TSC will have cystic disease on MRI, with it being common for some patients to have a solid mass associated with cystic components. Such lesions should be serially measured and assessed for growth characteristics to help distinguish fat-poor AMLs from the malignant AMLs (67,68).

### ADPKD

**Figure 3:**
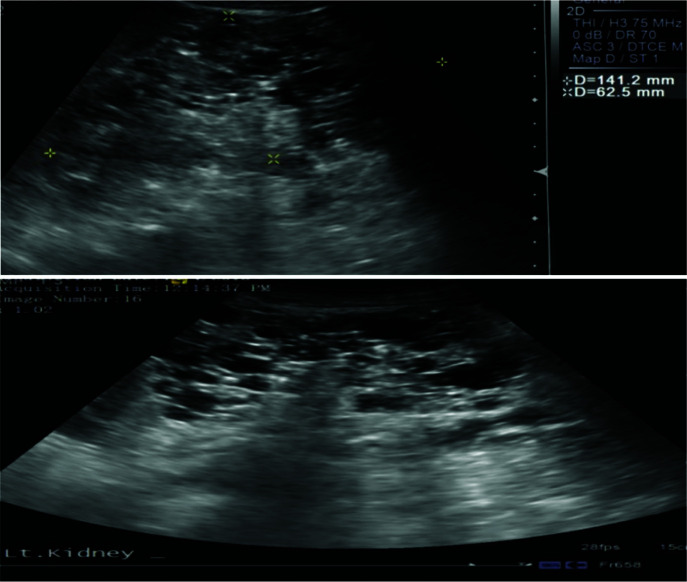
Ultrasound imaging reveals that both kidneys are enlarged with multiple cysts and loss of normal architecture, suggestive of polycystic kidney disease.

Polycystic kidney disease is characterized by the presence of an innumerable number of cysts that eventually occupy the renal parenchyma, culminating in renal failure and premature secondary hypertension. In most cases, diagnosis is established by utilizing clinical criteria; however, molecular genetic testing can be utilized for prenatal diagnosis, for screening family members of affected individuals, and for cases where diagnosis is ambiguous. According to the kidney-related surveillance and management recommendations by the International Tuberous Sclerosis Complex Consensus Group, MRI is the preferred modality for detection of renal cysts of ADPKD and TSC-associated AML, which is supplemented with frequent blood pressure screening and glomerular filtration rate (GFR) estimation (11). Renal US can be used to screen for AMLs and to monitor cysts; however, US can miss AMLs that are fat deprived or kidneys with numerous cysts. Thus, MRI of the kidneys may be preferable for clearer images (69). Figure 3 shows how a positive image of renal cysts looks like on an US. Children >12 years and adults have higher potential of developing AML and, therefore, should undergo an MRI every 1–3 years. In cases of TSC where initial MRI has exclusively ruled out the presence of AML, annual follow-up with US is recommended with an MRI every 2–3 years (70). In addition, genetic testing is recommended for TSC and ADPKD patients when they are presented with an ambiguous diagnosis (71, 72).

### Cystic disease

TSC renal cystic disease is identified conventionally by MRI in approximately 50% of patients (73). The size of renal cysts in TSC renal disease can range from microscopic glomerulocystic disease to the polycystic renal phenotype found in the *TSC2/PKD1* contiguous gene syndrome. In most patients with TSC, renal cysts are asymptomatic with a limited number of cysts that are smaller (73, 74). However, similar to AML, their prevalence increases with age (33). These types of cysts do not require regular surveillance imaging, but images should still be taken, as these patients are at risk for AML as they age. The renal and nonrenal manifestations of cystic disease are outlined in Table 5.

**Table 5:** Cystic subtypes seen in TSC.

Cystic disease type	Characteristics	Complications associated
TSC Polycystic	A high frequency of mosaicism, and often these children have cystic disease on prenatal ultrasound.	Develops very significant disease by 2 months of age. Hypertension is often discovered by the second week of life but can take several months to develop. These children sometimes develop a urinary concentrating defect very early.
TSC Cortical Microcystic	Findings are subtle on MRI and can be missed on ultrasound.	Can develop into CKD stage 2 or 3 in the late teens or early 20s. Hypertension is not common until significant CKD develops
TSC Focal Cystic	Appears as a cluster of cysts. The developmental timing can be in utero and only become phenotypically expressed after acute kidney injury.	Once expressed, it can result in rapid cyst formation and increased risk of hemorrhaging.
TSC Cortical Cystic	Identified by cysts limited to the cortex and columns of Bertin. This cystic disease occurs early on and the cysts are remarkably uniform in size, at least during childhood	Present in large amounts and can be mistaken for glomerulocystic disease.
TSC Multicystic	Cysts can also be distributed throughout the cortical and medullary tissue and exhibit variable sizes with loss of corticomedullary differentiation.	Due to the variability of sizes, it can present varying degrees of risk from hemorrhaging.

Adapted from Mulders YM. Large deletion causing the TSC2-PKD1 contiguous gene syndrome without infantile polycystic disease. J Med Genet. 2003;40(2):2–4 and Consugar MB, Wong WC, Lundquist PA, Rossetti S, Vickie J, (47) Walker DL, Rangel LJ, et al. Characterization of large rearrangements in autosomal dominant polycystic kidney disease and the PKD1/TSC2 contiguous gene syndrome. Kidney Int. 2009;74(11):1468–79. (75).

TSC, Tuberous sclerosis complex; MRI, magnetic resonance imaging; CKD, chronic kidney disease.

### Oncocytoma

Oncocytomas have been described in patients with TSC but seem to be generally rare and are almost always benign. Renal oncocytoma are histologically described as containing nests and tubular structures lined by cells with eosinophilic, granular cytoplasm (76). They are described as containing edematous myxoid or hyalinized stroma in some areas with tubular structures dispersed in the stroma. Clear cytoplasm may also be focally present with cells in lesions containing round nuclei and areas of degenerative cytologic atypia. These features can result in patches of tumor cells with large nuclei, irregular nuclear contours, and smudged chromatin (12). They are usually unilateral and single amongst the general population but can present as multiple and bilateral in patients with TSC (77). While the significance of renal lesions in TSC is firmly established, little is known about predictable biomarkers. Renal oncocytomas are a more common cause of renal cell neoplasms in patients with TSC than in the general population (78). The diagnosis of renal oncocytoma should be confirmed by immunohistochemistry to avoid differential diagnosis with epithelioid AMLs.

## Hypertension

Hypertension is a common secondary renal manifestation of TSC. Renal ischemia, growth of AML, and hyper secretion of renin can lead to an increase in blood pressure, independent of family history (75). A study conducted by Kingswood et al. on TSC patients in the United Kingdom showed that the prevalence of hypertension was 23% amongst children aged <18 years and 11% amongst adults aged >18 years (75). Hypertension in TSC patients was secondary to renal involvement in the form of cysts and scarring, which predisposes to hypertensive crisis in these patients. If treatment is not appropriately provided, hypertension can accelerate renal scarring and loss of GFR. This high prevalence and consequence of improper management prompts the requirement of an effective strategy for blood pressure control in these patients. Since cystic changes in the kidney contribute to elevated blood pressure, the use of ACE-inhibitors or angiotensin receptor blockers (ARB) is recommended (15). These drugs prevent the production of Angiotensin-II and counteract aldosterone action, resulting in a decrease in blood pressure in addition to the prevention of remodeling effects of elevated pressure on the kidneys and heart (15).

### Treatment

The first line of treatment for AMLs are the use of mTOR inhibitors given their ability to simultaneously reduce growth volume while preserving kidney function. In the presence of large lesions, it is preferable to undergo treatment through a pharmacological route as they often require extensive or repeat embolization, which can increase the risk of chronic kidney disease (CKD) (79). The modification of the upregulated mTOR pathway has been investigated in a growing number of clinical studies. The mTOR inhibitor, everolimus, is primarily used for patients who have multiple large angiolipomas that show evidence of ≥5 mm of growth per year and for patients who have had prior nephrectomy or embolization (44). With regard to AMLs, the first study demonstrating an antiproliferative effect of mTOR inhibitors was conducted in 20 patients. The results showed a significant reduction in AML size (80). The beneficial effects subsided once treatment stopped, indicating a reversible effect of mTOR inhibition. The EXIST-1 trial (n = 117) reported that 49% of patients had ≥50% reduction in volume after a mean of 9.6 months of treatment with everolimus (71). The Exist-2 trials found that after 24 weeks, 55% of patients receiving treatment showed >50% reduction in AML volume on 10 mg/day of everolimus. Furthermore, AML vascularization was reduced with everolimus treatment, providing an even more protective benefit through reduced risk of aneurism. These results led to the approval for the use of everolimus in the United States and Europe (81).

A similar drug, known as Sirolimus, is also very effective at significantly reducing the size of AMLs. Similar to everolimus, the protective effects were lost when treatment was discontinued. For both treatment routes, the most frequent side effects were stomatitis, headache, hypercholesterolemia, urinary tract infection, and amenorrhea, specifically in female patients (77). Furthermore, caution should be exercised in patients who have reduced renal function with estimated glomerular filtration rate (eGFR) of <45 mL/min/1.73 m^2^ at the onset of treatment. These patients are at risk for reductions in clearance, particularly if they also present with proteinuria. If mTOR inhibitors are used for such patients, the eGFR should be assessed within 1 month of starting treatment and every 4 months thereafter (82). Preclinical studies have shown that mTORC1 can contribute to the normal development of glomeruli and podocytes, while resulting in glomerular injury when decreased (82). Even kidneys with large renal AMLs can contribute significantly to renal function and should be treated primarily through pharmacological means. Nephrectomy and embolism should be considered primarily in emergent situations in order to avoid any unnecessary renal damage (82). A summary of mTOR inhibitor trials are shown in Table 6.

**Table 6:** List of mTOR inhibitors trials.

Study	N	Study protocol	Study outcome	Complications
Bissler et al. 2008 (70)	118	Double-blind, placebo-controlled, phase 3 trial; patients aged 18 years or older with at least one angiomyolipoma of 3 cm were chosen to receive oral everolimus 10 mg per day or placebo.	Response rate was 42% (33/79) >50% reduction in the growth of angiomyolipoma.	The most common adverse events in the everolimus and placebo groups were stomatitis (48%), nasopharyngitis (24%), and acne-like skin lesions (22%).
Davies et al. 2011 (83)	16	Multicenter phase 2 nonrandomized open label trial. Dosage given to achieve steady-state blood level of 3 to 10 ng/mL.	Angiomyolipoma size was reduced in all 16 patients and by 30% or more in eight patients.	The most common side effects seen were oral mucositis (6 of 16 patients), respiratory infections (5 patients), and proteinuria (5 patients.
Dabora et al. 2011 (84)	36	Daily sirolimus 10 mg/day.	Overall response rate was 44.4%; 16/36 had a partial response. Mean decrease in kidney tumor size was 29.9% at week 52.	Adverse effects that occurred at a frequency of >20% included stomatitis, hypertriglyceridemia, hypercholesterolemia, bone marrow suppression (anemia, mild neutropenia, leucopenia), proteinuria, and joint pain.
Kingswood et al. 2013 (15)	44	4.5 mg/m^2^/day everolimus to achieve target blood trough: 5–15 ng/mL.	Angiomyolipoma response rates were 53.3% (16/30) in the everolimus arm.	Mouth ulceration, convulsion, stomatitis, fatigue and rash were the most common adverse effects with everolimus therapy, with rates of 43%, 30%, 26.7%, and 20%, respectively.
Franz et al. 2012 (71)	117	Double-blind, placebo-controlled, phase 3 trial; patients were given oral everolimus 4•5 mg/m (2) per day (titrated to achieve blood trough concentrations of 5–15 ng/mL) or placebo.	27 (35%) patients in the everolimus group had at least 50% reduction in the volume of subependymal giant cell astrocytomas.	The most common adverse events were mouth ulceration (32%) in the everolimus group, stomatitis (31%), and convulsion (23%).
Bissler 2013 (81)	20	Subjects will resume the dosing regimen that they were receiving at the completion of the initial RAD001 study (Bissler 2008).	N/A, results not posted Primary Outcome: RAD001 tolerance	N/A
Lopez (2012) (85)	17	Four-month, prospective open-label, single-arm study. Initial dose of rapamycin (1 mg/d). Doses were increased by 1 mg every 2 weeks until stable plasma levels (4–8 ng/mL) were achieved.	58.8% (10/17) patients successfully had 50% AML volume reduction. After 6 months, mean volume decrease was 55.18%, and 1 year it was 66.38%.	No serious adverse effects were seen with oral aphthous ulcers (29.4%), hypertriglyceridaemia (29.4), diarrhea (17.6%), acneiform rash (11.8%), with microcytosis and hypochromia (17.6%) being the mild adverse effects seen.

mTOR, mammalian target of rapamycin; AML, angiomyolipoma.

## New and Experimental Therapies

While mTOR inhibitors alone have shown to be effective treatment options, second-generation therapy options are being developed in order to improve efficacy and clinical outcomes. One example is that of dual PI3K/mTOR inhibitors. Currently, rapamycin inhibitors work as incomplete inactivators of mTORC1. However, they can simultaneously stimulate feedback activation of PI3K/Akt mitogenic pathways (86). Dual P13K/mTOR inhibitors work as competitive inhibitors that bind to the ATP-binding site on the catalytic site of both mTORC1/2 and PI3K, which are two crucial signaling hubs (87). New dual PI3K/mTOR inhibitors have been developed and pushed into phase 1 and 2 trials. However, in spite of the potential, clinical studies thus far have shown limited efficacy and significant concern of adverse effects, including nausea, diarrhea, vomiting, hyperglycemia, cutaneous rash, elevated liver enzyme levels, renal failure, and hypertension (86). Further trials will need to be conducted to establish the limit of their use and the appropriate stage of treatment at which they should be introduced.

### ATP-competitive inhibitors: TOR-KIs

Second-generation mTOR inhibitors that directly target and block the ATP catalytic site of both mTOR complexes are being developed. This results in widespread inhibition of the mTOR signal (88). These drugs are found to be more specific than the current inhibitors due to lower half-maximal inhibitory concentration (IC_50_) against mTOR activity than PI3K (88). The specificity allows for reduced risk of adverse effects associated with dual PI3K/mTOR inhibitors (86). In cell models, TOR-KIs have also shown to be effective antiproliferators against cells with insensitivity to first-generation mTOR inhibitors (25). Multiple TOR-KIs have undergone clinical trials with regard to neoplastic disorders. Sadly, mechanisms of resistance have been observed for this class as well in part due to the ability of PI3K/Akt/mTOR pathway to adjust in the face of change to individual components (86).

### New generation: RapaLink-1

The third generation of drugs, known as RapaLink-1, is being developed in order to improve the efficacy, and to reduce resistance mechanisms and the incidence of side effects. By binding both the adenosine triphosphate (ATP) and the FKBP-rapamycin-binding (FRB) binding sites of mTOR, RapaLink-1 combines the strong affinity qualities of rapamycin for mTORC along with the kinase-inhibiting abilities of TOR-KI (89, 90). A polyethylene glycol unit connects the rapamycin-binding domain to FKBP12 along with the FRB domain of mTOR. This interaction leads to high selectivity of rapamycin to mTORC1 along with the kinase-inhibiting domain MLN0128 binding to the ATP site of mTORC1. Rapalink-1 has been found to be effective at inhibiting mTORC1 and mTORC2 downstream targets at doses as low as 1 and 3 nM in early clinical trials. While the full mechanism has not been elucidated, early results show its effectiveness in suppressing catalytic activity mTORC components (86).

### Prognosis of renal manifestations of TSC

The prognosis of patients with TSC is dependent on the severity and scope of symptoms that they develop. All patients are at risk for life-threatening conditions related to brain tumors, kidney lesions, or LAM. Regardless of the stage and severity of the conditions, continued monitoring and imaging is crucial for developing an appropriate treatment plan. Appropriate medical intervention can allow most patients to have normal life expectancy with minimal loss of function. In rare cases, infections or tumors in vital organs can lead to severe issues and death. Patients with medium (4–8 cm) and large (>8 cm) AMLs are most at risk for injury or death (91). In these situations, early initiation of mTOR treatment can reverse growth and lessen the strain on the renal system and secondary organ systems. In a retrospective study by Amin et al., the majority of deaths in TSC were attributed to renal issues. Of those that died due to renal issues, 62.5% died due to hemorrhaging of AMLs or RCC. The remaining patients died due to complications from CKD and polycystic kidney disease. While this study was conducted after the widespread use of mTOR inhibitors, the clinical studies mentioned previously show that their continued use can improve the expected prognosis of these patients and mitigate the loss of renal function (61).

### Future perspective

In terms of future research, long-term studies are necessary to entirely ameliorate the long-term renal effects of mTOR inhibitors. While current safety profiles do not project a large percentage of serious adverse effects, long-term maintenance needs to be studied in order to better understand if changes in dosage must be made. Furthermore, more research must be done in elucidating the full pathogenesis of the TSC pathway to better understand the effects of hormones on the development of AMLs and how this may account for the difference in manifestation between male and female presentation of AMLs. More research into gene therapy might also be the method to restore function in the absence of pharmacological therapy or a potential cure.

## Conclusion

TSC is a systemic and/or organ-specific disorder that manifests on the kidney, heart, lungs, and brain in the form of skin lesions. Proper understanding of the *TSC1/2* pathway and how its downstream effects lead to the development of tumors are paramount for understanding the pathogenesis of TSC and for developing new techniques for treating this disorder. An understanding of the renal manifestations, current treatment, and diagnosis can allow physicians to better tackle these cases when they arise.
